# Bee Venom Alleviates Atopic Dermatitis Symptoms through the Upregulation of Decay-Accelerating Factor (DAF/CD55)

**DOI:** 10.3390/toxins11050239

**Published:** 2019-04-26

**Authors:** Yenny Kim, Youn-Woo Lee, Hangeun Kim, Dae Kyun Chung

**Affiliations:** 1 Graduate School of Biotechnology, Kyung Hee University, Yongin 17104, Korea; 5033743@naver.com (Y.K.); younwoo1093@naver.com (Y.-W.L.); 2 Skin Biotechnology Center, Kyung Hee University, Yongin 17104, Korea

**Keywords:** Bee venom, complement system, decay accelerating factor, atopic dermatitis, complement dependent cytotoxicity, membrane attack complex

## Abstract

Bee venom (BV)—a complex mixture of peptides and toxic proteins including phospholipase A2 and melittin—promotes blood clotting. In this study, we investigated the anti-atopic properties of BV and the mechanism associated with its regulation of the complement system. BV treatment upregulated the mRNA and protein levels of CD55 in THP-1 cells. Further experiments revealed that the phosphorylation of ERK was associated with upregulation of CD55. A complement-dependent cytotoxicity assay and a bacteria-killing assay showed that BV inactivated the complement system through the induction of CD55. The serum levels of C3 convertase (C3C) and Membrane attack complex (MAC) increased, while CD55 decreased in mice with AD-like lesions from DNCB treatment. However, the levels were inverted when the AD-like mice were treated with BV using subcutaneous injection, and we observed that the AD symptoms were alleviated. BV is often used to treat AD but its mechanism has not been elucidated. Here, we suggest that BV alleviates AD through the inactivation of the complement system, especially by the induction of CD55.

## 1. Introduction

Bee venom (BV) is a secretion from the stinger of the worker bee; it is a complex mixture of proteins they use to protect themselves. Purified BV from honeybees has been used as a traditional medicine by the ancient Egyptians, Chinese, and Greeks [1]. BV contains pharmaceutically active peptides including melittin, apamin, adolapin, and the mast cell degranulating (MCD) peptide; enzymes (e.g., phospholipase A2, PLA2); biologically active amines (e.g., histamine and epinephrine); and nonpeptide components [2]. Melittin, the major component (50% of dry weight) of BV, has anti-inflammatory and anti-arthritis properties, which are driven by the inhibition of nuclear factor kappa B (NF-κB) [3]. Melittin has shown anticancer, antibacterial, and antiviral activities [4]. PLA2 from BV improved atopic dermatitis (AD)-like skin lesions induced by dust mite extract in mice. Topical application of PLA2 suppressed AD symptoms, including ear thickness, histological changes, inflammatory cytokines, and serum IgE concentration [5]. The anti-inflammatory effects of BV are expected to improve skin inflammatory diseases such as AD, but this has not been clearly demonstrated.

AD is the most common allergic skin disease, but its pathogenesis is complex and still not fully understood. Researchers have shown that the complex immune reactions associated with the Th2 response and IgE production affect AD, but Th22, Th17, and Th1 activation also occur in AD [6]. In addition, the complement system seems to affect AD. One study reported that complement components including C3, C4, and C3a are increased in AD patients compared to non-atopic controls [7,8]. Overactivation of the complement system has been shown to cause damage to the dermal-epidermal junction [9], which may aggravate AD. In addition, the anaphylatoxin C5a receptor is increased in AD mice, and treatment with a C5aR antagonist decreased IL-4 and IFN-γ levels in skin tissue, as well as the levels of IL-4, IFN-γ, histamine, and IgE in the serum, indicating that blocking C5aR can inhibit AD [10]. However, the role of complement inhibitory proteins (CIPs) in AD has not been elucidated.

In the current study, we investigated the role of BV in the regulation of the complement system. We examined the upregulation of CD55 in THP-1 cells and the levels and activities of C3 convertase (C3C) and membrane attack complex (MAC) from the serum of AD-like mice after treatment with BV or melittin. 

## 2. Results

### 2.1. BV Increased CD55 Production in THP-1 Cells

**Figure 1:**
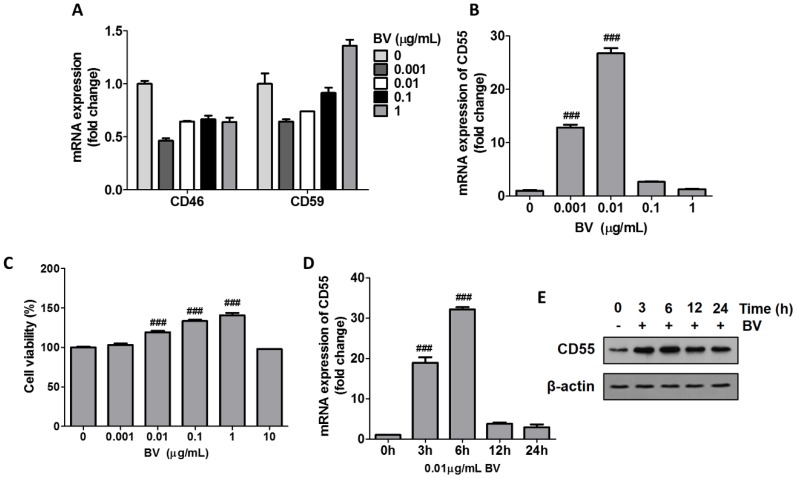
Bee venom (BV) increased CD55 production in THP-1 cells. (**A**) THP-1 cells were treated with the indicated doses of BV for 6 h. The mRNA levels of CD46 and CD59 were examined by qRT-PCR. (**B**) After BV treatment with the indicated doses for 6 h, CD55 mRNA was examined by qRT-PCR. (**C**) Cell viability was examined by WST-1 assay with THP-1 cells treated with the indicated dose of BV for 24 h. (**D**) THP-1 cells were treated with 0.01 μg/mL BV for the indicated time periods. Levels of mRNA were normalized with glyceraldehyde 3-phosphate dehydrogenase (GAPDH). The data are displayed as the mean ± SD of three independent experiments. Statistical analysis was conducted with one-way ANOVA Tukey statistical test. ### *p* < 0.001 compared to 0 μg/mL or 0 h. (**E**) THP-1 cells were treated with 0.01 μg/mL BV for the indicated times and CD55 protein was examined by Western blot. β-actin was used as the internal control. Data are representative of three independent experiments.

Because complement cascades are regulated by membrane-bound complement regulators including membrane cofactor protein (MCP/CD46), decay-accelerating factor (DAF/CD55), and CD59 [11], we investigated whether THP-1 cells express cell membrane CIP in response to BV. CD46 mRNA was reduced by approximately 50% in the cells after stimulation with BV, while the mRNA levels of CD59 were reduced by a low level of BV (e.g., 0.001–0.1 μg/mL) and slightly upregulated by 1 μg/mL BV (Figure 1A). However, there were no statistically significant differences between the samples. These results suggested that BV-induced variations in CD46 and CD59 may not affect the activation of the complement system and AD symptoms. CD55, unlike other CIPs, showed a bell-shaped curve when cells were stimulated with BV. The highest induction of CD55 was caused by 0.01 μg /mL BV, and higher dosages of BV such as 0.1 and 1 μg/mL reduced CD55 expression compared with untreated cells (Figure 1B). When cells were treated with BV doses of up to 10 μg/mL, cell death was not observed, indicating that the induction and inhibition of CD55 by BV in cells were not associated with cell survivability (Figure 1C). Actually, the viability of THP-1 cells was significantly increased by BV (0.01–1 μg/mL), suggesting that BV may affect cell proliferation. When cells were treated with 0.01 μg/mL BV, CD55 mRNA peaked at 6 h and then declined (Figure 1D). The protein levels of CD55 also increased from 3 h after stimulation and peaked at 6 h; they then declined after 12 h but were still higher than in unstimulated cells (Figure 1E). Our data suggest that BV regulates CD55 expression in immune cells, which may affect complement cascades in the bloodstream. 

### 2.2. BV Induced CD55 Through the Activation of ERK

**Figure 2:**
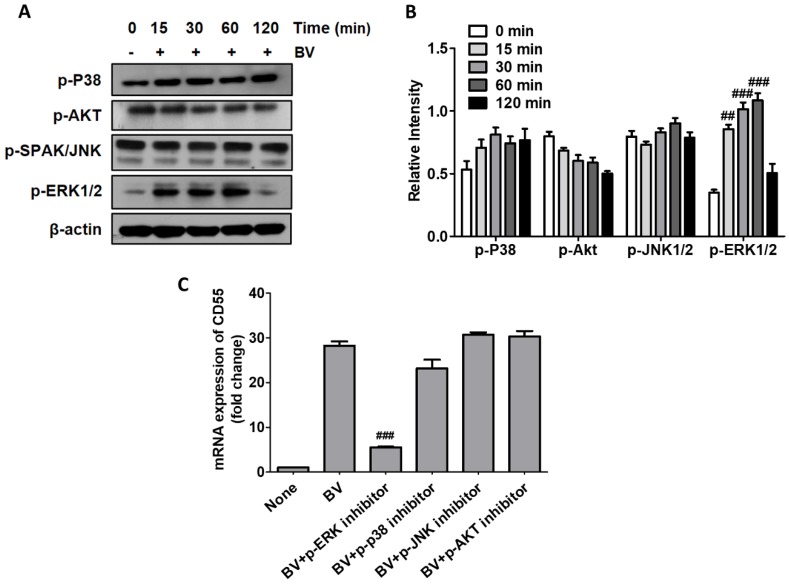
The extracellular signal regulated kinase (ERK) signaling pathway was associated with CD55 induction in THP-1 cells. (**A**) THP-1 cells were treated with 0.01 μg/mL BV for the indicated times and the phosphorylated signaling factors were examined by Western blot. β-actin was used as the internal control. The data are representative of two independent experiments. (**B**) Densitometry analysis for the phosphorylation of p38, Akt, JNK1/2, and ERK1/2. (**C**) THP-1 cells were treated with the signaling inhibitors for ERK, p38, JNK, and Akt for 30 min and then treated with 0.01 μg/mL BV for 6 h. The mRNA levels of CD55 were examined by qRT-PCR. Levels of mRNA were normalized with GAPDH. Data are displayed as the mean ± SD of three independent experiments. Statistical analysis was conducted with one-way ANOVA Tukey statistical test. ## *p* < 0.01; ### *p*< 0.001 compared to none or 0 min.

Next, we investigated the signaling pathway related to the BV-mediated induction of CD55 in THP-1 cells. After treatment with BV, activation of extracellular signal regulated kinases (ERKs) was observed, while other signaling molecules, including P38 mitogen-activated protein kinase (p38), protein kinase B (Akt), and c-Jun N-terminal kinases (JNK1/2), were not altered (Figure 2A). The densitometry analysis also indicates that the phosphorylation of ERK1/2 was activated by BV (Figure 2B). We examined the phosphorylation of NF-κB subunit p65, but it was not activated by BV treatment (data not shown). When cells were pretreated with the inhibitors for each signal, only the ERK inhibitor reduced CD55 expression in BV-treated cells, indicating that BV increases CD55 expression through the activation of ERKs in THP-1 cells (Figure 2C).

### 2.3. BV Alleviated AD Symptoms

**Figure 3:**
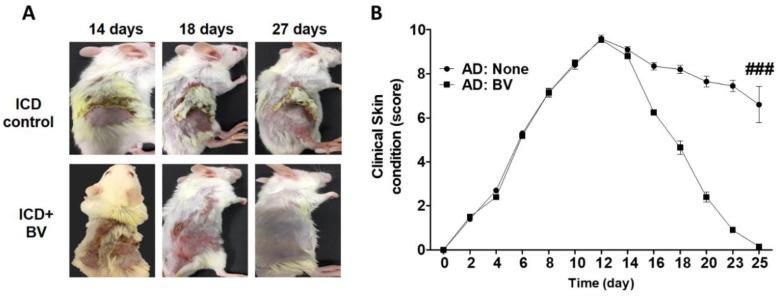
BV alleviated AD symptoms. An AD-like condition was induced in mice with 2.5% (*w*/*v*) DNCB for 3 days followed by 1% (*w*/*v*) DNCB at 3-day intervals. After 14 days of treatment with DNCB, one group (*n* = 4) was subcutaneously injected with 0.3 mg/kg BV and the other group (*n* = 4) was injected with PBS. Both groups were treated with 0.2% (*w*/*v*) DNCB to prevent spontaneous remission. (**A**) A photograph of the mice with the best effect among the experimental groups is shown. A representative mouse from each group is shown. (**B**) The severity of AD was quantified by individually scoring the symptoms (skin dryness, hemorrhage, edema, redness, and excoriation). Data are displayed as the mean ± SD of technical repeats of one representative experiment. We performed two independent experiments. Statistical analysis was conducted with two-way ANOVA Tukey statistical test. ### *p* < 0.001 compared to the none group.

Since BV has been used as traditional medicine [1], we examined whether BV has a treatment effects of AD. An AD-like condition was induced in mice (irritant contact dermatitis (ICD)) by treatment with 2.5% 2,4-Dinitrochlorobenzene (DNCB) for 14 days. The experimental group was subcutaneously injected with BV and treated with 0.2% DNCB, while the control group was treated only with 0.2% DNCB. Clinical assessment of the ICD mice was performed as described in the Materials and Methods section. The skin condition such as dryness, hemorrhage, excoriation, edema and redness, increased significantly in the DNCB-treated mice (ICD) at 14 days, but it was attenuated in the group that was injected with BV (ICD+BV) at 18 days and the skin almost completely recovered at 27 days (Figure 3A). The clinical skin score in the BV-injected group also significantly decreased at 12 days as compared to the control group (Figure 3B).

### 2.4. BV Inactivated Complement System in AD-Like Mice 

**Figure 4:**
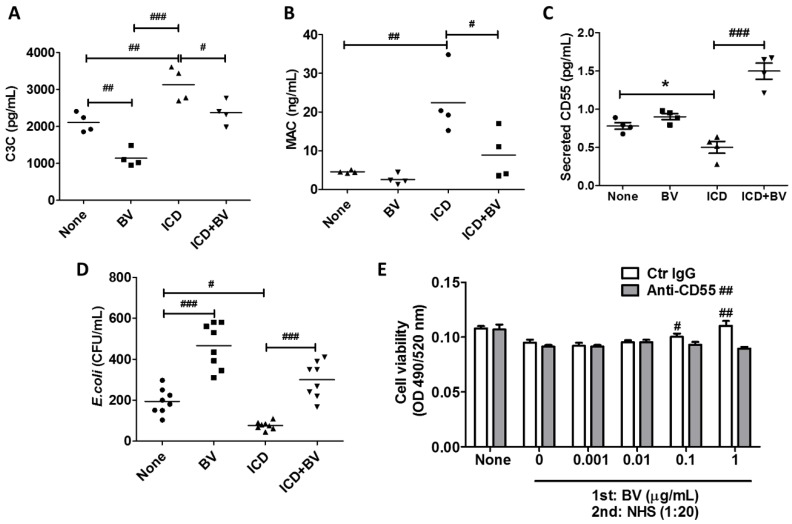
BV injection reduced the activities of C3C and membrane attack complex (MAC) through the induction of CD55. Blood samples were taken from AD-like mice (*n* = 4, each group) after 0.3 mg/kg BV injection, and serum was isolated. (**A** and **B**) Serum levels of C3C and MAC were examined by commercial ELISA kits. (**C**) The serum CD55 levels were examined by indirect ELISA using an anti-CD55 antibody. (**D**) The bactericidal activity of complement was examined with sera isolated from each group. (**E**) A complement-dependent cytotoxicity assay was performed with normal human serum (NHS) in HaCaT cells treated with 0.01 μg/mL BV for 24 h in the presence or absence of anti-CD55 antibody. Cell viability was examined by Calcein-AM (a fluorogenic, cell-permeant fluorescent probe) assay. NHS was isolated from blood supplied by the Blood Center of the Korean Red Cross. Data are displayed as the mean ± SD of four independent experiments. Statistical analysis was conducted with an unpaired two-tailed *t* test (**C**). * *p* < 0.05 compared to none, one-way ANOVA Tukey statistical test, # *p* < 0.05; ## *p* < 0.01; ### *p* < 0.001 compared between indicated groups (A to D) or compared to 0 μg/mL (E) or Two-way ANOVA, ## *p* < 0.01 compared between Control IgG and Anti-CD55.

We examined whether BV affects complement in an AD-like mouse model, developed by treatment with 2.5% DNCB. First, we measured the levels of C3C and MAC in serum using sandwich ELISA kits. The serum levels of C3C and MAC in normal mice significantly decreased after BV treatment. In mice that developed ICD, both levels were significantly higher compared with the untreated mice (‘none’ in Figure 4A–D). When comparing the ICD mice groups, both C3C and MAC significantly decreased after BV injection compared with PBS injection (Figure 4A,B). On the other hand, the secreted CD55 serum levels decreased in ICD mice, but significantly increased after BV injection (Figure 4C). These data suggest that BV inhibits the complement system. The actual activity of complement was examined in mouse serum. The bactericidal activity decreased with BV in untreated mice, while it increased in ICD mice compared to the untreated mice. The bactericidal activity in ICD mice decreased in the BV-injected ICD mice (Figure 4D), indicating that MAC activity was increased in AD, which is consistent with previous studies [7,8], and it was decreased by BV injection. Since an abnormal increase of MAC damages skin tissues, it is necessary to maintain an appropriate level of MAC. Thus, BV may be a good candidate to maintain MAC homeostasis. Next, we examined the role of CD55 in complement-mediated tissue damage, using a complement-dependent cytotoxicity assay (Figure 4E). Normal human serum (NHS, 1:20 dilution) significantly decreased HaCaT cell viability, indicating that complement induced the cell death of keratinocytes. However, when NHS was added to the BV-treated cells, the viability increased in a BV dose-dependent manner. Increased viability, however, was not shown when cells were treated with anti-CD55 neutralization antibody prior to BV treatment, indicating that increased CD55 inhibits MAC activity, which may affect the alleviation of AD symptoms.

### 2.5. Melittin Alleviated AD Symptoms Through the Regulation of Complement

**Figure 5:**
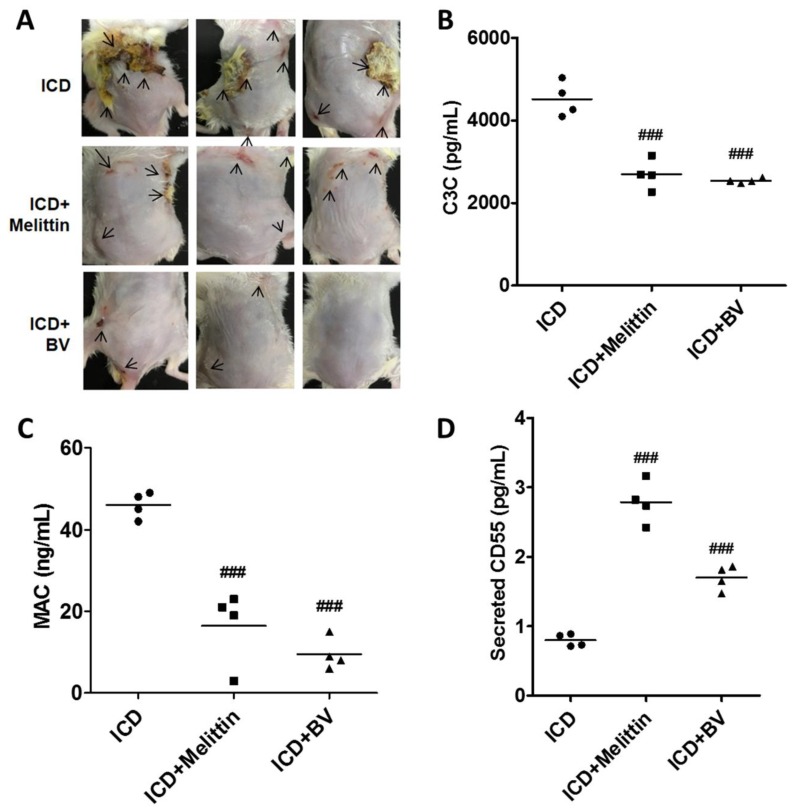
Melittin plays an important role in the BV-mediated alleviation of AD symptoms. An AD-like mouse model (*n* = 4, each group) was generated with 2.5% DNCB, and mice were subcutaneously injected with 0.3 mg/kg BV or 0.15 mg/kg melittin. (**A**) A photograph of the AD-like mice is shown after 14 daysof treatment with BV or melittin. Serum C3C (**B**) and MAC (**C**) levels were examined by commercial ELISA kits. (**D**) Secreted CD55 was examined by indirect ELISA with the sera isolated from BV- or melittin-treated mice and normal mice. Data are displayed as the mean ± SD of three independent experiments. Statistical analysis was conducted with one-way ANOVA Tukey statistical test. ### *p* < 0.001 compared to ICD. Arrows indicate AD-like skin condition (i.e., hemorrhage and excoriation, and edema and redness).

When ICD mice were subcutaneously injected with melittin (0.15 mg/kg), AD symptoms were alleviated as seen in BV-injected mice. Melittin is a major component of BV and contributes anti-inflammatory and anticancer effects [2,4]. Skin condition was improved in melittin-injected ICD mice as well as in BV-injected ICD mice (Figure 5A). To examine whether melittin affects complement, C3C levels were examined in serum from ICD mice that were injected with melittin. As shown in Figure 5B, serum C3C levels were significantly lower compared with untreated ICD mice. The same reduction was shown in BV-injected mice. MAC levels in ICD mouse serum were also significantly decreased in melittin-injected mice compared to the ICD control mice, although they were slightly higher than in the BV-injected mice (Figure 5C). The secreted CD55 levels in melittin-injected ICD mice were significantly higher than in the control ICD mice, suggesting that the complement system was reduced (Figure 5D). These data suggest that BV, especially melittin, can alleviate AD via controlling complement.

## 3. Discussion

Purified BV has anti-inflammatory and anticancer effects [12]. It also reduces AD symptoms, lowering serum IgE levels and dorsal skin thickness [13]. AD is a chronic skin inflammatory disease characterized by eczematous, dry, and chapped skin. AD is caused by the invasion of inflammatory immune cells including mast cells, eosinophils, monocytes/macrophages, and T lymphocytes into the skin barrier. The circulating eosinophils and serum IgE levels are increased in AD, which is associated with interleukin (IL)-4, IL-5, and IL-13 produced by the Th2 cells in most patients [14,15,16,17,18]. In addition, complement appears to aggravate AD. Patients with AD show increases in complement components including C3, C4, and C3a [7,8]. Because activation of complement generates a high level of MAC, MAC can worsen AD lesions, aggravating AD symptoms. 

The complement system is an essential part of the immune system. It has long been described as belonging to the innate immune system, but recently a number of papers have demonstrated that it also contributes to adaptive immunity by regulating antigen-presenting cells [19]. As an inducer for innate immunity, MAC, a final product of complement cascades, kills invading bacteria, and anaphylatoxins, such as C3a and C5a, activate inflammatory responses. However, excessive activation of the complement system can induce tissue damage and inflammation, which aggravates symptoms in patients with AD [20,21]. Thus, it is necessary to reduce excessive activation of the complement system and restore homeostasis to defend against bacterial infection. Toxins isolated from insects and snakes seem to have beneficial effects against inflammatory diseases [22], but they are also toxic and potentially life-threatening for mammals [23]. 

BV has beneficial effects on idiopathic Parkinson’s disease and oxaliplatin-induced neuropathic cold allodynia, and it is helpful in reducing glutamate-induced cell toxicity in neurodegenerative diseases [24,25,26,27]. BV components, especially melittin, inhibit complement cleavage and release bradykinin. These mechanisms are associated with coagulation, thrombolysis, hemolysis, and smooth muscle tone [28]. Recently, Shaldoum et al. have reported that BV may affect complement system. According to the results, in patients having various diseases, such as rheumatoid arthritis, back pain, diabetes mallets, arthritis, gastritis, sebaceous cyst, osteoarthritis, and hepatitis c virus, all abnormal levels of complement C3 returned to normal values, while abnormal C4 levels did not change when patients were exposure to natural BV [29]. In this study we have established the following mechanisms of BV in the alleviation of AD; (i) BV induced CD55 production through the activation of ERK1/2 pathways; (ii) increased CD55 downregulated formation of C3C and MAC; (iii) decreased MAC activity resulted in the alleviation of AD symptoms. BV may be a promising drug to treat AD, because it inhibits complement by inducing CD55. Among CIPs, only CD55 dramatically increased in BV-treated THP-1 cells and in serum from BV-injected mice. Although the mechanism for CD55-dependent inactivation of C3C is complex [30], the expected result is a reduction in MAC formation. The dose-dependent variation of CD55 in Figure 1B suggests that BV has different activities in mammalian cells. BV causes the activation of the immune system, which could increase the symptoms of atherosclerosis, diabetes-related endothelial damage, cancer, and autoimmune diseases [31]. However, it also has anti-inflammatory properties and is used in the treatment of liver fibrosis, atherosclerosis and other skin diseases [32].

We found that CD55 did not completely inhibit C3C in BV-injected mice. As shown in Figure 3, the increased C3C in BV-injected mice was restored to a normal state. The MAC level and activity also recovered to a normal state. These data suggest that BV does not inhibit the complement system completely, but can still protect against invasion of bacteria and support the repair of damaged tissues. However, the appropriate dosage should be considered when applied to mammals. The complement inhibitory effects of BV and melittin suggest that they can be used to treat complement-mediated diseases such as ischemia/reperfusion injury and autoimmune disorders, which are caused by excessive complement activation [21]. 

In conclusion, BV, especially melittin, appears to alleviate AD. This phenomenon appears to be mediated by ERK pathway activation leading to the induction of CD55 in BV-treated cells. CD55-mediated inhibition of complement alleviates AD symptoms, which can otherwise be aggravated by inflammation and MAC. Thus, BV can be considered as a therapeutic reagent to treat AD as well as inflammatory diseases. 

## 4. Materials and Methods

### 4.1. Cell Culture 

HaCaT and THP-1 cells were maintained with Dulbecco’s modified Eagle’s medium (DMEM, Welgene, Gyeongsangbuk-do, Korea) and RPMI 1640 (Welgene), respectively, supplemented with 10% (*v*/*v*) heat-inactivated fetal bovine serum (FBS, Welgene) and 1% (*v*/*v*) penicillin–streptomycin (P/S, Welgene). These cells were incubated at 37 °C in a CO_2_ atmosphere.

### 4.2. Drugs

Bee venom was purchased from Guju Pharmaceutical Company, Ltd. (Gyeonggi-do, Korea). Bee venom was dissolved in Dulbecco’s phosphate-buffered saline (DPBS). 

### 4.3. Real-Time PCR

For real-time PCR, total RNA was extracted using TRIzol reagent (Takara Bio Inc., Shiga, Japan), following the manufacturer’s instructions. cDNA was synthesized using an iScript cDNA synthesis kit (Bio-Rad, San Diego, CA, USA), following the manufacturer’s instructions. CFX Connect™ Real-Time PCR Detection System (Bio-Rad) and SYBR Ex TaqTM^II^ (Takara) were used for real time PCR. The following forward and reverse primers were used; 5′-GTGAGGAGCCACCAACATTT-3′ and 5′-GCGGTCATCTGAGACAGGT-3′ for CD46; 5′-CAGCACCACCACAAATTGAC-3′ and 5′-CTGAACTGTTGGTGGGACCT-3′ for CD55; 5′-CCGCTTGAGGGAAAATGAG-3′ and 5′-CAGAAATGGAGTCACCAGCA-3′ for CD59; and 5′-AAGGTCGGAGTCAACGGATT-3′ and 5′-GCAGTGAGGGTCTCTCTCCT-3′ for GAPDH. The target gene expression was normalized with glyceraldehyde-3-phosphate dehydrogenase (GAPDH). The contamination of Mycoplasma was examined with EZ-PCR™ Mycoplasma Detection Kit (Catalog # SKU:20-700-20; Biological Industires, Cromwell, CT, USA) and we found no contamination.

### 4.4. Western Blot

THP-1 cells treated with BV (0.01 µg/mL) were lysed with Laemmli buffer and boiled for 5 min at 100 °C. Proteins were separated by 10% (*w*/*v*) or 12% (*w*/*v*) SDS-PAGE in a Glycine/Tris/SDS buffer and transferred onto polyvinylidene fluoride membranes for 2 h at 100 V. The membranes were blocked with 5% (*w*/*v*) bovine serum albumin in TBST (20 mM Tris-HCl, 150 mM NaCl, 0.05% (*v*/*v*) Tween 20) for 2 h at room temperature (RT) and washed three times with TBST. The membrane was incubated with the primary antibodies such as anti-phospho p38 (#9211), anti-phospho Akt (#9271), anti-phospho SPAK/JNK (#9251), anti-phospho ERK1/2 (#9101) (Those were purchased from Cell Signaling Technology Inc., Danvers, MA, USA), and β-actin (SC47778, Santa Cruz Biotechnology, Inc., Dallas, TX, USA) diluted in TBST (1:1000) for 2 h at RT and then washed with TBST three times. Next, the membrane was incubated with secondary HRP-conjugated anti-rabbit or anti-mouse antibody (diluted to 1:2000 in TBST) for 2 h at RT. After washing three times with TBST, the bands were detected by ECL reagent. β-actin was used as the internal loading control. 

### 4.5. Animals 

Male BALB/c mice (7 weeks old) were purchased from Nara Bio (Gyeonggi, Korea). They were kept in individual cages at 24 ± 2°C and 50 ± 10% moisture, and fed nutritionally balanced rodent food (Central Lab Animal Inc., Seoul, Korea) and sterilized water. The mice were cared for and used in accordance with the guidelines of the Animal Ethics Committee of Kyung Hee University (KHU14-021, the date of the approval: 26 October 2015).

### 4.6. Development of Irritant Contact Dermatitis (ICD) Mouse

Mice were shaved on the dorsal flank and back. They were left for 24 h to heal any abrasions that might have been caused by shaving. Olive oil and acetone were mixed at a ratio of 1:3, and then DNCB was added to make concentrations of 2.5% (2,4-dinitrochlorobenzene; Sigma-Aldrich Co., St. Louis, MO, USA), 1.0%, and 0.2%. Mice were topically treated with the 2.5% (*w*/*v*) DNCB mixture (200 μL). After 3 days exposure, mice were treated with 150 μL 1.0% (*w*/*v*) DNCB at 3-day intervals until 14 days, and then were treated with the 0.2% (*w*/*v*) DNCB mixture (100 μL). 

### 4.7. Treatment of ICD Mice Using BV

**Figure 6:**
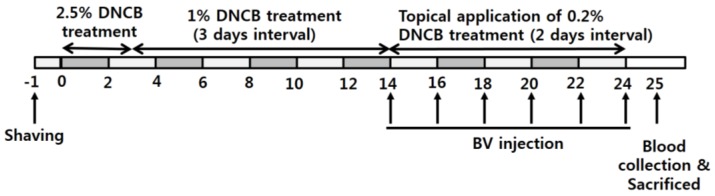
Time schedule for ICD generation, BV injection, and blood collection.

The mice were randomly divided into four experimental groups of four animals each as follows (Figure 6): untreated normal group (None), BV-treated normal group (BV), untreated ICD group (ICD), and BV-treated ICD group (ICD+BV). The BV and ICD+BV groups were subcutaneously injected with 0.3 mg/kg BV at 2-day intervals, and None and ICD groups were subcutaneously injected with 60 μL PBS. For the analysis of serum complement components, blood samples were collected at day 25 before sacrifice. The blood samples were maintained at room temperature for 20 min. Then, the serum was separated by centrifugation at 12,000 rpm for 20 min. The serum samples were used to examine the MAC and C3C quantities and the bacterial killing assay.

### 4.8. Clinical Skin Score.

Mice in each experimental group were photographed using a digital camera to analyze AD symptoms and the clinical appearance of the skin. AD symptoms were evaluated by scoring scaling and dryness, hemorrhage and excoriation, and edema and redness. The sum of the individual symptom scores was calculated (0 = normal, 1 = mild, 2 = moderate, 3 = severe). The total score for each animal ranged from 0 to 9 points.

### 4.9. Enzyme-Linked Immunosorbent Assay (ELISA) 

The quantities of MAC and C3C were measured in mouse serum. The MAC and C3C ELISA kits were purchased from MyBioSource (San Diego, CA, USA). The assay was performed according to the manufacturer’s instructions. The absorbance was measured at the wavelength of 450 nm using an ELISA reader. The concentrations of MAC and C3C were calculated using the standard included in the kits. For the detection of soluble CD55 from mouse serum, rabbit anti-CD55 (Santa Cruz, CA, USA) was coated on an ELISA plate (Corning Costar flat-bottom high-binding EIA/RIA 3690 plate) in PBS (pH 7.4; 0.4 µg/well) at 37 °C for 2 h. The plate was blocked with 2% BSA in PBS. Mouse serum was added in triplicate to experimental or control wells. After incubation at 4 °C overnight, wells were washed and bound CD55 was detected by serial addition of a biotin-labeled secondary antibody, avidin–horseradish peroxidase (HRP) conjugate (Pierce Immunopure streptavidin-HRP conjugate), and peroxidase substrate (Pierce Chemicals, IL). The absorbance at 450 nm was read and presented after subtraction of reagent-control values reacting against BSA-coated negative controls.

### 4.10. Complement-Dependent Cytotoxicity Assay

HaCaT cells were seeded on a 96-well plate with DMEM supplemented with 10% FBS and P/S. The cells were pretreated with BV together with a control IgG or an anti-CD55 neutralization antibody (Santa Cruz Biotechnology, SC51733, Dallas, TX, USA) and washed with DPBS. Normal human serum (NHS) was diluted 1:20 with DMEM without supplement, and the cells were incubated for 6 h. The cells were washed with DPBS and the viability of the HaCaT cells was measured with the Calcein AM cell viability assay system (EMD Millipore, #206700, Burlington, MA, USA). Briefly, 2 µM Calcein AM (final concentration) was added to each well and incubated for 15 min at 37 °C under CO_2_. Fluorescence was examined at at 490 nm excitation and 520 nm emission wavelengths.

### 4.11. Bactericidal Assay

*Escherichia coli* was cultured in LB overnight. Then, the bacteria were washed and diluted with DPBS. *Escherichia coli* at 1 × $10^{4}$ cells was cultured with mouse serum (1:50) at 37 °C for 60 min. The incubated bacteria were washed with DPBS and spread on an LB plate. After overnight culturing, CFU were counted.

### 4.12. Statistical Analysis

All the experiments were repeated at least three times. The data shown are representative results of the means ± SD of triplicate experiments. Statistical analyses were conducted with an unpaired two-tailed *t*-test, then one-way ANOVA, followed by Tukey’s honestly significant difference (HSD) post hoc test, or two-way ANOVA. Prism 5 software was used for the analysis (Graphpad software Inc., Prism 5 (Version 5.01, San diego, CA, USA, 2007). *p* < 0.05 was considered significant. The * represents the *t*-tests while the # represents the ANOVA in the figures.

## References

[1] Hoyt J. (2015). Literature Review of Bee Venom Therapy: Mechanisms of Action and Selected Therapeutic Uses. Orient. Med. J..

[2] Lariviere W.R., Melzack R. (1996). The bee venom test: A new tonic-pain test. Pain.

[3] Lee G., Bae H. (2016). Anti-Inflammatory Applications of Melittin, a Major Component of Bee Venom: Detailed Mechanism of Action and Adverse Effects. Molecules.

[4] Raghuraman H., Chattopadhyay A. (2007). Melittin: A membrane-active peptide with diverse functions. Biosci. Rep..

[5] Jung K.H., Baek H., Kang M., Kim N., Lee S.Y., Bae H. (2017). Bee Venom Phospholipase A2 Ameliorates House Dust Mite Extract Induced Atopic Dermatitis Like Skin Lesions in Mice. Toxins.

[6] Gittler J.K., Shemer A., Suárez-Fariñas M., Fuentes-Duculan J., Gulewicz K.J., Wang C.Q.F., Mitsui H., Cardinale I., de Guzman Strong C., Krueger J.G. (2012). Progressive activation of Th2/Th22 cytokines and selective epidermal proteins characterizes acute and chronic atopic dermatitis. J. Allergy Clin. Immunol..

[7] Kapp A., Wokalek H., schöpf E. (1985). Involvement of complement in psoriasis and atopic dermatitis-measurement of C3a and C5a, C3, C4 and C1 inactivator. Arch. Dermatol. Res..

[8] Kapp A., Schöpf E. (1985). Involvement of complement in atopic dermatitis. Acta. Derm. Venereol. Suppl. (Stockh).

[9] Zhuang Y., Lyga J. (2014). Inflammaging in skin and other tissues—the roles of complement system and macrophage. Inflamm. Allergy Drug Targets.

[10] Dang L., He L., Wang Y., Xiong J., Bai B., Li Y. (2015). Role of the complement anaphylatoxin C5a-receptor pathway in atopic dermatitis in mice. Mol. Med. Rep..

[11] Noris M., Remuzzi G. (2013). Overview of Complement Activation and Regulation. Semin. Nephrol..

[12] Son D.J., Lee J.W., Lee Y.H., Song H.S., Lee C.K., Hong J.T. (2007). Therapeutic application of anti-arthritis, pain-releasing, and anti-cancer effects of bee venom and its constituent compounds. Parmacol. Ther..

[13] Gu H., Kim W.H., An H.J., Kim J.Y., Gwon M.G., Han S.M., Leem J., Park K.K. (2018). Therapeutic effects of bee venom on experimental atopic dermatitis. Mol. Med. Rep..

[14] Leung D.Y., Guttman-Yassky E. (2014). Deciphering the complexities of atopic dermatitis: Shifting paradigms in treatment approaches. J. Allergy Clin. Immunol..

[15] Schlapbach C., Simon D. (2014). Update on skin allergy. Allergy.

[16] Lim S.J., Kim M., Randy A., Nam E.J., Nho C.W. (2016). Effects of Hovenia dulcis Thunb. extract and methyl vanillate on atopic dermatitis-like skin lesions and TNF-α/IFN-γ-induced chemokines production in HaCaT cells. J. Pharm. Pharmacol..

[17] Galli S.J., Tsai M., Piliponsky A.M. (2008). The development of allergic inflammation. Nature.

[18] Owen C.E. (2007). Immunoglobulin E: Role in asthma and allergic disease: Lessons from the clinic. Pharmacol. Ther..

[19] Killick J., Morisse G., Sieger D., Astier A.L. (2018). Complement as a regulator of adaptive immunity. Semin. Immunopathol..

[20] Tsokos G.C., Fleming S.D. (2004). Autoimmunity, complement activation, tissue injury and reciprocal effects. Curr. Dir. Autoimmun..

[21] Markiewski M.M., Lambris J.D. (2007). The Role of Complement in Inflammatory Diseases From Behind the Scenes into the Spotlight. Am. J. Pathol..

[22] Sales T.A., Marcussi S., da Cunha E.F.F., Kuca K., Ramalho T.C. (2017). Can Inhibitors of Snake Venom Phospholipases A₂Lead to New Insights into Anti-Inflammatory Therapy in Humans? A Theoretical Study. Toxins.

[23] Harris J.B., Scott-Davey T. (2013). Secreted Phospholipases A2 of Snake Venoms: Effects on the Peripheral Neuromuscular System with Comments on the Role of Phospholipases A2 in Disorders of the CNS and Their Uses in Industry. Toxins.

[24] Doo K.H., Lee J.H., Cho S.Y., Jung W.S., Moon S.K., Park J.M., Ko C.N., Kim H., Park H.J., Park S.U. (2015). A Prospective Open-Label Study of Combined Treatment for Idiopathic Parkinson’s Disease Using Acupuncture and Bee Venom Acupuncture as an Adjunctive Treatment. J. Altern. Complement Med..

[25] Lee J.H., Li D.X., Yoon H., Go D., Quan F.S., Min B.I., Kim S.K. (2014). Serotonergic mechanism of the relieving effect of bee venom acupuncture on oxaliplatin-induced neuropathic cold allodynia in rats. BMC Complement Altern. Med..

[26] Lim B.S., Moon H.J., Li D.X., Gil M., Min J.K., Lee G., Bae H., Kim S.K., Min B.I. (2013). Effect of bee venom acupuncture on oxaliplatin-induced cold allodynia in rats. Evid. Based Complement Alternat. Med..

[27] Lee S.M., Yang E.J., Choi S.M., Kim S.H., Baek M.G., Jiang J.H. (2012). Effects of bee venom on glutamate-induced toxicity in neuronal and glial cells. Evid. Based Complement Alternat. Med..

[28] Mingomataj E.C., Bakiri A.H. (2012). Episodic hemorrhage during honeybee venom anaphylaxis: Potential mechanisms. J. Investig. Allergol. Clin. Immunol..

[29] Shaldoum F.M., Hassan M.I., Hassan M.S. (2018). Natural Honey Bee venom Manipulates Human Immune Response. Egypt J. Hosp. Med..

[30] Janeway C.A., Travers P., Walport M., Shlomchik M.J. (2001). Immunobiology: The Immune System in Health and Disease.

[31] Park J.H., Yim B.K., Lee J.H., Lee S., Kim T.H. (2015). Risk associated with bee venom therapy: A systematic review and meta-analysis. PLoS ONE.

[32] Lee W.R., Pak S.C., Park K.K. (2015). The protective effect of bee venom on fibrosis causing inflammatory diseases. Toxins.

